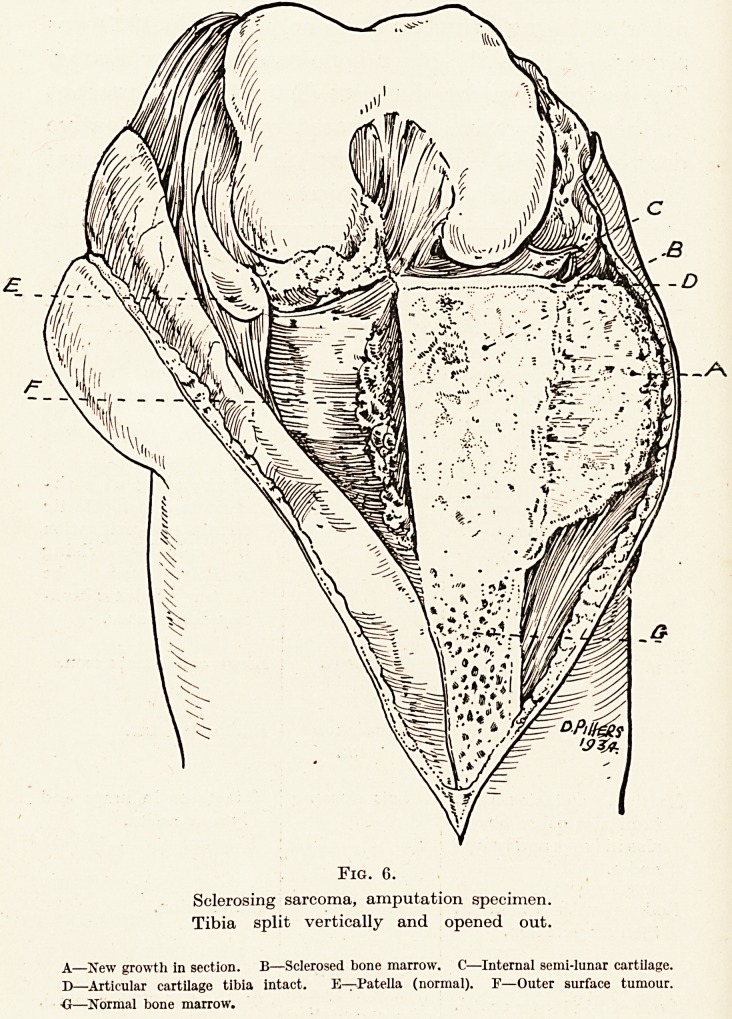# The Diagnosis of Swellings of the Long Bones, with a Case of Periosteal Sarcoma of the Femur
*A Paper read at a Meeting of the Bristol Medico-Chirurgical Society on Wednesday, 11th April, 1934.


**Published:** 1934

**Authors:** A. Wilfrid Adams

**Affiliations:** Senior Assistant Surgeon, Bristol Royal Infirmary; Surgeon, Bristol Children's Hospital


### THE DIAGNOSIS OF SWELLINGS OF THE LONG BONES, WITH A CASE OF PERIOSTEAL SARCOMA OF THE FEMUR.*

BY

A. Wilfrid Adams, M.S., F.R.C.S.,

*Senior Assistant Surgeon, Bristol Royal Infirmary;*
*Surgeon, Bristol Children's Hospital.*

---

This communication owes its inspiration to the repeated anxieties which the author has felt when trying to elucidate the nature of bony swellings. The problem seemed almost insoluble in the case of femoral sarcoma now reported. It recurs frequently among the cases cited as illustrations of the differential diagnosis. In the handling of this every-day difficulty the patient's good and the reputation of the medical profession are seriously at stake.

### Osteogenetic Sarcoma of the Femur.

### Case Report

M. N., a female, 9 years old, came into the Bristol Children's Hospital in June, 1931, complaining of pain in the right hip for one month; there had been a previous attack lasting a fortnight a year before. Examination revealed a tender fulness which could be seen and felt below the great

* A Paper read at a Meeting of the Bristol Medico-Chirurgical Society on Wednesday, 11th April, 1934.

trochanter of the right side. The circumference of the thigh was $\frac{7}{8}$ in. less than that of the left side. She walked with a slight limp.

Skiagraphy, a month previously, had shown the cortex of the right femur, below the great trochanter, to be evenly thickened to twice the thickness of that of the left side. On a skiagram taken 14th June, 1931, Dr. Mayes reported : " Old inflammatory condition of great trochanter of right femur. The shaft shows periostitis . . . the condition appears to be quiescent." (Fig. 1A.)

*Operation.*—An exploratory operation 16th June, 1931, revealed an unduly cancellous bone mixed with a jelly-like material.

*Biopsy Report.*—" The appearance suggested to my mind the possibility of remote trauma, with subsequent repeated hæmorrhages and insufficient absorption. There are a few blood-vessels, however, showing intimal changes and collars of monocytic cells."

Septic discharge followed the operation for one week, but the wound healed well and after three weeks she left the hospital walking well.

Pain recurred, however, and disturbed her rest. A further examination showed an obvious, spindle-shaped swelling below the great trochanter. This was again explored on 29th September, 1931, and specimens sent to two pathologists.

*Report A.*—" Shows evidence of simple chronic inflammation."

*Report B.*—" I find little that is abnormal. There is certainly no evidence of neoplasm."

During the following autumn slight pyrexia and pain continued, and the circumference of the limb increased in size from 14 in. (12th November, 1931,) to 15 in. (10th December, 1931) despite two injections of colloidal selenium given on 18th and 25th November, 1931. The Wassermann reaction was negative, even after a provocative injection of novarsenobillon. The only abnormality found in a differential blood-count was a rise in small lymphocytes to 42 per cent. As she was well in other respects and the disease was progressing a diagnosis of sarcoma became compulsory. I was glad to have the help of Mr. Harry Platt's opinion at this juncture, and with his encouragement to amputate.

PLATE I.

FIG. 1A.

Femur, sarcoma (M.N., 9 years).

FIG. 1B.

Femur (M.N.), section after removal.

### PLATE II.

Fig. 2.

Humerus, septic periosteal node and abscess.

Fig. 3.
Radius, syphilitic osteitis.

Fig. 4.
Femur, osteolytic sarcoma with spontaneous fracture.

Fig. 5.
Radius, sclerosing sarcoma.

*Operation.*—15th December, 1931. Under a spinal anæsthetic, disarticulation at the hip, followed by a blood transfusion. Satisfactory convalescence. Pathological report on specimen after operation: "Sclerosing osteogenetic sarcoma."

*Present condition.*—11th April, 1934: She is a happy, bonny girl, with no signs of recurrence.

Before considering the special problem of the foregoing case it is useful to take a wide survey of the whole subject of this paper. To clarify the study a simple pathological classification has been adopted.

### DIFFERENTIAL DIAGNOSIS.

1. *Traumatic Osseous Deposits.* — Among the swellings on a long bone those due to trauma provide many traps for the unwary. In the foregoing case there was a vague history of injury, and the smooth, osseous node below the great trochanter might have passed for traumatic periostitis. Indeed, one distinguished London surgeon did so diagnose the skiagraphic picture. In another instance a hard mass at this very site proved to be of a simple nature.

A docker, aged about 50 years, came some weeks after a blow on the hip with a hard, tender area the size of a child's fist and arising rather abruptly from the right femoral shaft just below the great trochanter. Skiagraphy showed bony flecks in the soft parts, overlying the femur. Fortunately, though bruising was no longer visible by the time I saw him, the history of injury was sufficiently clear to justify the belief that there was no osteo-sarcoma but myositis ossificans present, and time confirmed this.

The occasional occurrence of sarcoma following a clearly-established history of bony injury increases the need for caution in giving an opinion. A fear of sarcoma may arise from a swelling due to a slow growth of callus around the site of an ununited crack across the tibia, especially if the patient first consults his doctor many months after injury. The delusion is quickly dispelled by radiography and the disability by a walking plaster.

The tendency for myositis ossificans to follow particular injuries often greatly aids diagnosis. For instance, when a hard mass develops after fracture or dislocation at the elbow and skiagraphy shows a bony shadow along the front of the humerus, there is no need to think twice about the underlying nature of the trouble. Again, the common tender and hard swellings that develop in front of the thigh after severe injuries to muscle or bone give rise to little anxiety as regards their pathological significance. They are apt to correspond in extent to the anatomical outlines of the quadriceps, and long strips of bone seen in the lateral skiagram parallel to the femoral shaft are characteristic. With rest their reduction slowly becomes apparent.

In regard to the nomenclature of this condition Watson Jones and Roberts[1] offer valuable criticism. They state that the term "myositis ossificans" is misapplied to these traumatic osseous deposits. They are really due to ossification in hæmatomata formed between the bone and the periosteum that has been stripped up. The correct name is "ossified periosteal hæmatoma," while myositis ossificans should be reserved for cases in which myositis is present.

In doubtful cases of sarcoma it may be necessary to resort to exploration. Such biopsies are very responsible undertakings. The weighty decision for or against amputation may have to be made more on the naked-eye evidence at this time than on the later histological findings, which are liable to be inconclusive in bone tumours. Boyd[2] quotes Ewing's warning on bone biopsies, which states: "The more experienced the pathologist the more he learns to rely on clinical data for clinical diagnosis, and the more he urges the surgeon to make his diagnosis on clinical observation and not to expect too much from the study of small pieces of tissue."

2. *Sepsis.*—As a rule septic infection gives distinctive evidence of its presence, yet sarcoma may mimic it. Thus the case of M. N. shared some of the stigmata usual in sepsis, such as a trace of fever and temporary discharge following exploratory operation, but no leucocytosis was present. On rare occasions sarcoma may closely simulate even acute pyogenic invasion of bone.

In 1923 a lad of 15 years was sent urgently by his doctor with a shin that was red and tender. The leg had troubled him for a month, and for a week he had had severe pain and a temperature of 100°. Acute infective periostitis seemed certain from the appearance.

I incised the periosteum and explored the bone, but found no obvious disease, nor did the pathologist's report on the bone-marrow help. It stated: "The tissue is bone and marrow; the marrow shows hæmorrhages and excess of polynuclear leucocytes. A few doubtful gram positive cocci are present; they are too few for certainty."

Two rigors occurred during his stay of a month. Four months later he returned after terrible suffering with a considerable increase in the size of the limb and a discharging sinus. Sarcoma suggested itself, for on exploration the tissue had a ground glass appearance and cut like gristle. Still the pathologist reported : " Chronic irritation, somewhat suspicious of syphilis." Some surgeons even at this advanced stage had favoured a diagnosis of chronic infective osteitis. On excavating deeper, bony tissue was removed, and after examining this the pathologist reported : " Osteosarcoma."

The leg was amputated ; he worked comfortably for two years, and then recurrent pain and metastasis began, of which he died two years later.

The contrary may happen, and what suggests sarcoma to the doctor may happily turn out to be sepsis.

A mother of 35 years, shortly after the birth of her baby, developed a hard swelling difficult to define deep on the lower end of the femur with mild fever. There was a history of injury in infancy and a small scar, but the woman had known no trouble since. The skiagram showed a small area of rarefaction in the adjacent femur.

At operation a few ounces of pus escaped, and a surprisingly extensive sequestrectomy of the lower third of the bone was needed.

This case also usefully illustrates the astonishing period of latency associated with osteomyelitis. The skiagram of L. M. (Fig. 2) affords an example of a dense surface node developed around a pyogenic infection in subperiosteal bone. Local excision cured it.

Owing to a septic metastasis in bone a spontaneous fracture may occur, and when the clinical picture corresponds with that in sarcomatous cases it may for a time mystify the doctor.

A man of 28 years broke the shaft of the right humerus by merely holding the rail of a tram car from which he was alighting in the usual way. Great swelling and discoloration was present when the arm was seen a day later and suggested a sarcomatous basis for the spontaneous fracture. The skiagram showed a break, but no disease.

In time the nature of the underlying lesion proved to be chronic pyæmia. He died eight months later from uræmia, after continual attendance for dressings of a chronic sinus. The post-mortem revealed multiple renal abscesses.

It is instructive to compare this with the following story :—

" A young woman attended my out-patient clinic with a fractured shaft of the humerus. A day or two before she felt her arm snap as she was getting over a stile. The skiagram showed nothing beyond a simple oblique fracture, but within a week the limb, instead of diminishing in the usual way, became increasingly swollen and painful. The condition was obviously sarcomatous. She refused amputation, and the arm was the size of a thigh before she died of pulmonary metastases months later."

3. *Tuberculosis.*—Tuberculosis, too, is often a close mimic of sarcoma, and both, like sepsis, are found most often at the ends of the bones in the active vascular area of the metaphysis.

When a child limps it may be only with difficulty and after two and three examinations that it is possible to assure oneself of the presence of a firm swelling in the bone adjacent to one of the joints. The skiagraphic evidence is apt to be slender and inconclusive at this equivocal stage, and may show a slight excavation or hint of periosteal roughening of the bone. The temperature is usually not raised, but there is help from the age incidence, for tuberculosis is frequent in the first decade, whereas sarcoma in a limb usually appears in adolescence or later. When faced with this common diagnostic dilemma the expectant line of treatment is best. Record the character of the swelling, circumference of the limb, and place it at rest. A tuberculous case usually declares itself either by amelioration of the symptoms or, if progressing, by softening of the swelling, the appearance of further foci about the skeleton, or obvious characteristics in subsequent skiagrams. Sometimes sarcomatous disease of the end of a long bone is called "chronic tuberculous arthritis." The mistake is as easy as it is deplorable to make. There is chronic, insidious pain followed by muscle wasting, stiffness of and a swelling so near the joint that the condition clinically is easily labelled "tumor albus."

4. *Syphilis.*—Gummatous osteitis and periostitis may bear a close resemblance to sarcoma owing to the severe chronic pain and swelling. Whereas with sarcoma the tumour rises abruptly from the shaft, in gumma the normal bone blends rather imperceptibly. The skiagram shows a less discrete localized disturbance than is usual with sarcoma. (Fig. 3.) In doubtful cases a Wassermann reaction is needed; and if positive should be followed by the therapeutic test with potassium iodide, for the patient may be the victim of both maladies.

5. *Swellings on Joints and Tendons.*—On two occasions bursitis over the upper end of the tibia has given rise to difficulty in diagnosis. In the first case, at operation, the negative skiagram was confirmed by the finding of a simple subsartorial bursa. This was in a seaman of some 50 years. In a girl of 17 years, with this bursa (as proved at a second operation), sarcoma of the underlying bone was the root of the trouble. Deceptive features in the latter case were (1) the ready withdrawal of 20 c.c.'s of typical clear synovial fluid which was found normal by the pathologist, and (2) after a first operation a biopsy report of " much inflammatory osteitis." A similar swelling in another lad, who was a chronic asthmatic invalid, was dissected out cleanly and proved to be tuberculous.

Fibro-sarcoma of a tendon sheath is sometimes found ominously hard and fixed as if arising from the bone itself. Twice I have met with such a mass, projecting from and as large as the medial condyle of the tibia, and once in connection with a finger flexor tendon. Osteo-arthritic nodules in the front of the hip-joint or subpsoas bursa provide deep-seated, hard swellings suggestive at times of sarcoma. A skiagram in the former case, or needle aspiration in the latter, reveal the truth of the matter.

6. *Non-sarcomatous Tumours of Bone.*—The foregoing are the genuine stumbling-blocks in the diagnosis of swellings on a long bone, and thanks to the superb radiography of to-day they are fewer than formerly. Osteoma, chondroma and solitary fibrocystic disease always have distinctive appearances in skiagrams, and from a practical point of view need seldom cause anxiety in diagnosis Solitary myeloma, whether giant or plasma-celled, tends to expand rather than project from the bone. The thinned shell of bone often breaks spontaneously and skiagraphy is typical. In diffuse myelomatosis it is to be noted that the disease may be confined to one tumour for the first year or two, and that the characteristic Bence-Jones proteosuria is absent in a considerable percentage. In a case of pathological fracture or paraplegia in older folk secondary malignant deposits need to be borne in mind.

7. *Rheumatism.*—Some cases have come to me labelled rheumatism or sciatica which have proved to be sarcomatous. Since in its early months a deep-seated tumour may give no evidence of swelling, but merely aches and pains, the condition may resemble rheumatism. The patient, too, usually has a healthy record and looks otherwise well, which further tempts the clinician to affix this common diagnostic label. Once such a conclusion is reached there is a dangerous tendency for it to put an end to fresh cogitation on the underlying cause when the patient returns for more of the medicine that has not yet cured. Therefore, when rheumatism resists medication and continues in one place, the doctor should be ever mindful of this most menacing possibility; mention of which seems justifiable, although pain and not bony swelling is the symptom concerned.

8. *Sarcoma.*—Proceeding to the study of sarcoma, as exemplified in the case reported, the clinician encounters the further complication of multiplicity of type of this tumour. As there are four varieties of this bone disease it is necessary to consider to which group the case belongs.

Ossification in the growth served to exclude two, namely parosteal sarcoma, whose origin is in the ensheathing fibrous layer of the periosteum, and the commoner osteolytic form (Fig. 4) arising in the osteogenetic cells. This latter causes porosity of bone, so that spontaneous fracture is common.

There remain the two types associated with a deposition of bone. One is known as the sclerosing form of osteogenetic sarcoma (Figs. 5 and 6), and the other as Ewing's tumour of bone, which in 1920 was segregated from the other bone sarcomata by Ewing. The justification for this new distinction is apparent in this comparative table based on the illuminative description of bone tumours in Geschickter and Copeland's book[3] on the subject.

| EWING'S SARCOMA. | | SCLEROSING OSTEOGENETIC SARCOMA. |
|---|---|---|
| 5–15 years. | *Age.* | 15–25 years. |
| Average thirteen months. General condition fair. Slight rise of temperature may occur. | *History.* | Average ten months. Marked wasting. |
| Thickening of the cortex due to laying-on of thin, dense layers of bone produces onion-peel appearance. | *Site.* | Thickening of sub-periosteal osteoblastic tissues and ossification in spicules, rising radially from the cortex produces sun-ray appearance. |
| Small round cells like lympho-sarcoma. | *Histology.* | Large osteoblasts seen. |
| Radio-sensitive. | *Irradiation result.* | Radio-resistant. |
| Multiple metastases in skull and other bones, also in lungs and lymph nodes. | *Late stages.* | Metastases in lungs and lymph nodes. |
| Reduce with radiation, then amputation. | *Treatment.* | Amputation. |
| 4 out of 24 (17 per cent.). | *Prognosis. Well at five years after amputation or excision.* | 16 out of 60 (27 per cent.). |

FIG. 6.

Sclerosing sarcoma, amputation specimen.
Tibia split vertically and opened out.

A—New growth in section. B—Sclerosed bone marrow. C—Internal semi-lunar cartilage. D—Articular cartilage tibia intact. E—Patella (normal). F—Outer surface tumour. G—Normal bone marrow.

Considering the case of M. N. in the light of this table it seems justifiable to classify her neoplasm as Ewing's tumour of the femur. On clinical examination the tumour was felt to rise gradually from the level of the unaffected bone, whereas characteristic in sclerosing sarcoma is the abrupt junction its uprising contour makes with the adjacent bone. (Fig. 6.) The skiagram (of a section prepared after operation) confirms this feature and shows a dense laminated deposit on the femur (Fig. 1B), and there are points, both in clinical and the earlier histological investigations, suggestive of infectious disease. Had I known of the therapeutic test based on its distinguishing radiosensitivity, deep X-rays would have been used to see if retrogression resulted. Such a response occurs notably in lymphatic disorders, which lends support to Kolodny's theory[3] of a lymphatic origin for Ewing's sarcoma.

The happy post-operative sequel so far has afforded no opportunity for further study as to the type of sarcoma in M. N. Subsequent history may yet throw light on the case, but despite the many suggestive clinical and naked-eye features that seem to favour Ewing's tumour, Professor Walker Hall (9th April, 1934) confirms the opinion that the case is histologically one of sclerosing sarcoma.

Certainly this case of sarcoma of the upper end of the femur, whatever its exact type, shows how great may be the value of amputation in this awful disease of young life.

Reviewing the literature, it seems reasonable to hope that with the help of our improving radiological evidence and a keener clinical effort amputation will not be marred by preliminary explorations and tedious investigations. Granted this improvement in diagnosis, the recurrence rate after amputation is likely to fall.

REFERENCES.

[1] Watson Jones and Roberts, "Calcification, Decalcification, Ossification," *Brit. Jour. Surgery,* 1934, xxi. 491.

* Boyd, *Surgical Pathology,* Saunders, Philadelphia, 1925, First Edition, p. 728.

[3] Geschickter and Copeland, *Tumors of Bone,* American Jour. of Cancer, New York, 1931.

## Figures and Tables

**Fig. 1a. f1:**
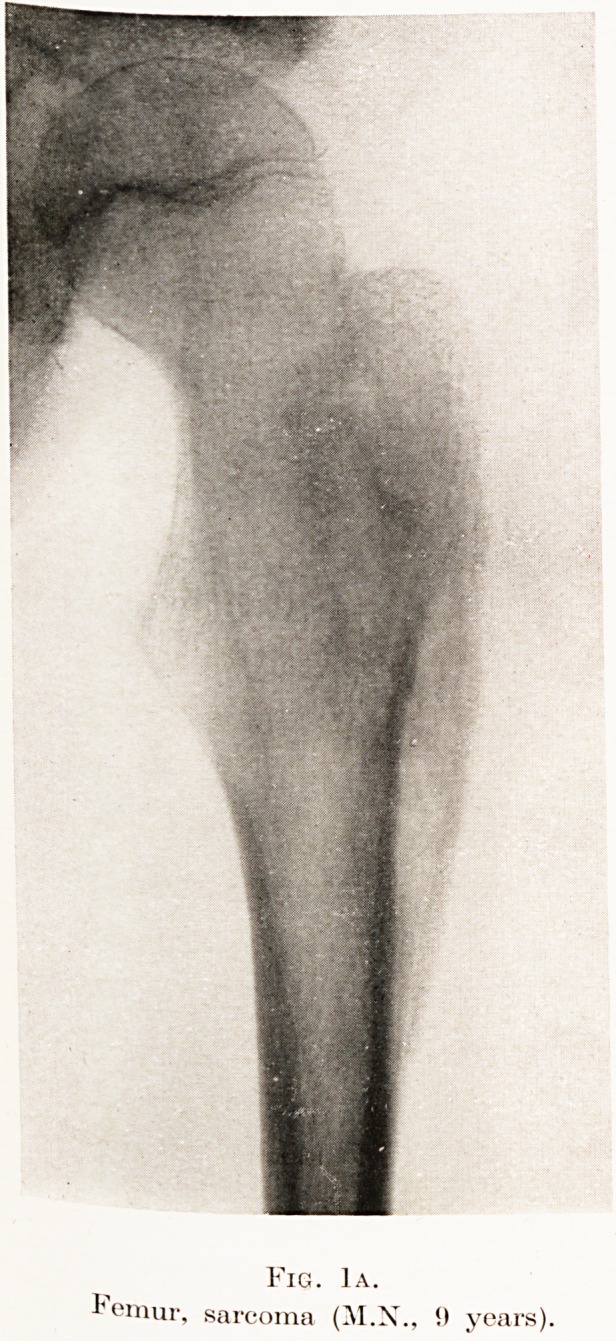


**Fig. 1b. f2:**
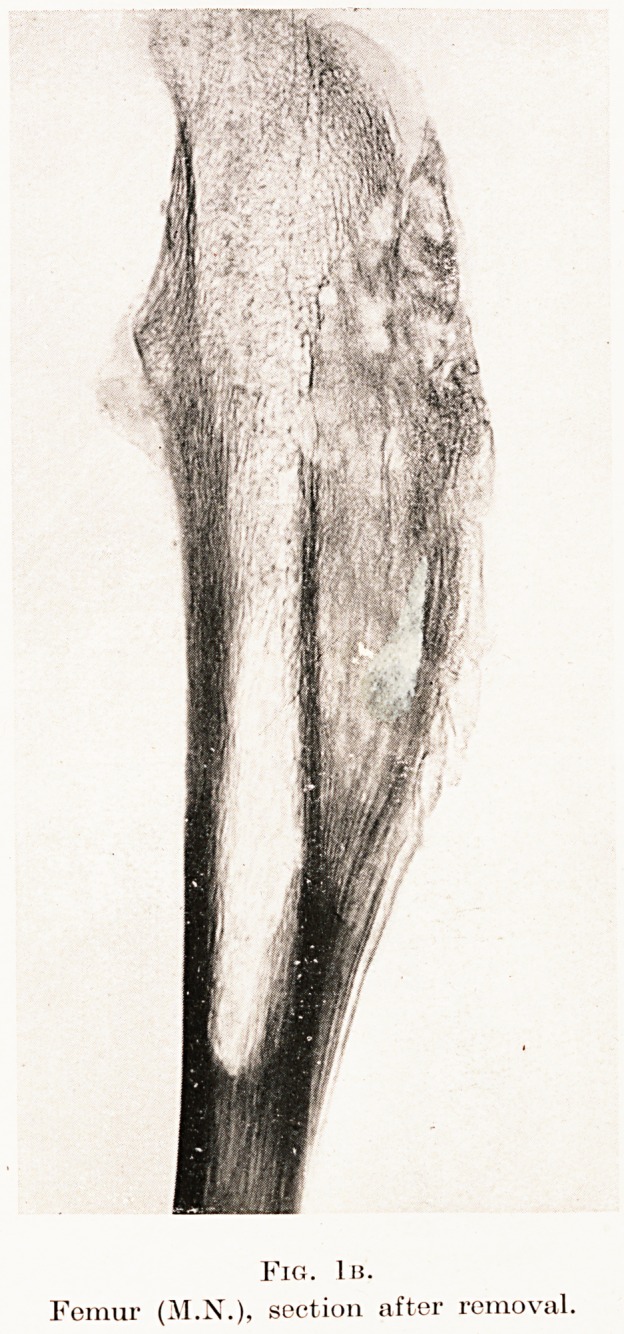


**Fig. 2. f3:**
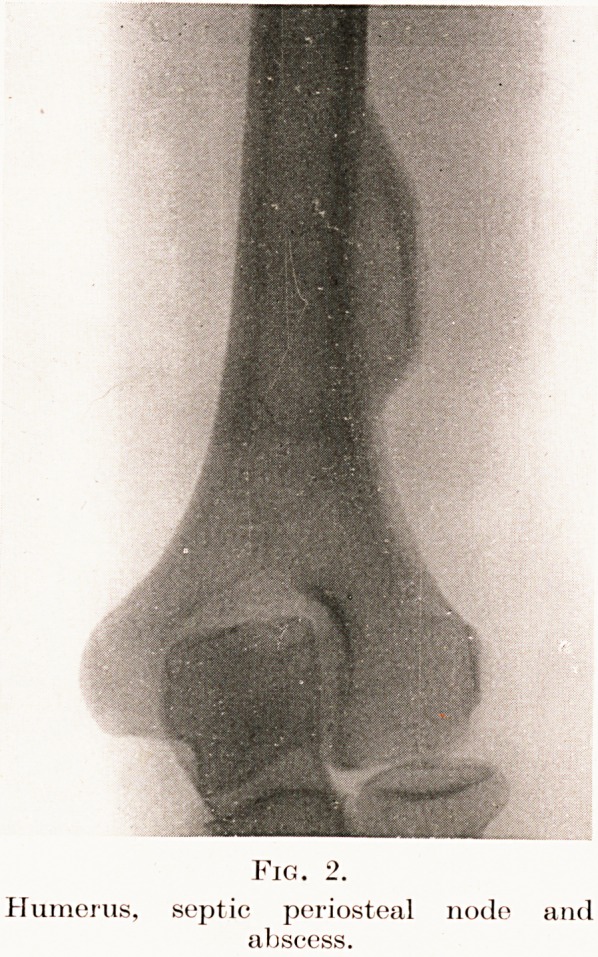


**Fig. 3. f4:**
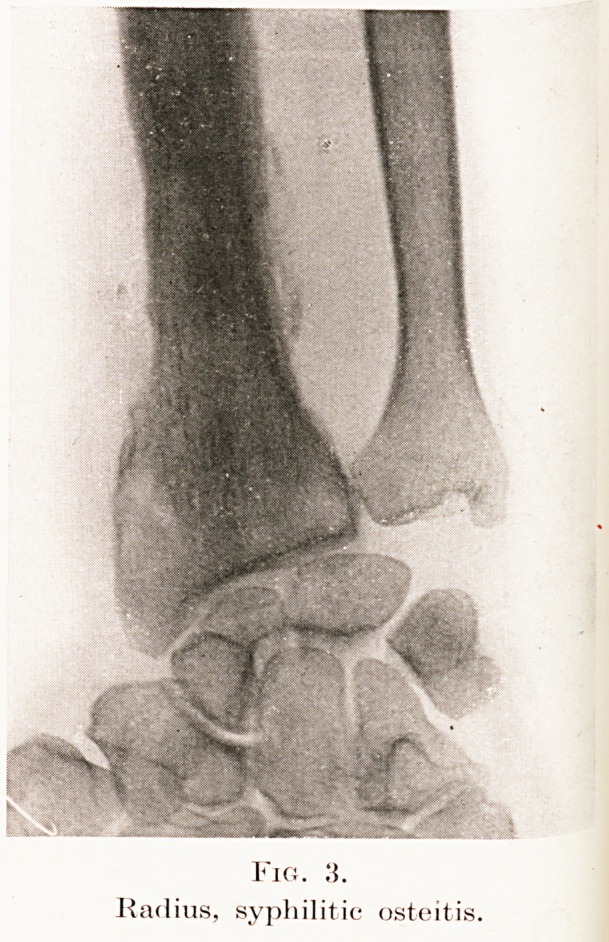


**Fig. 4. f5:**
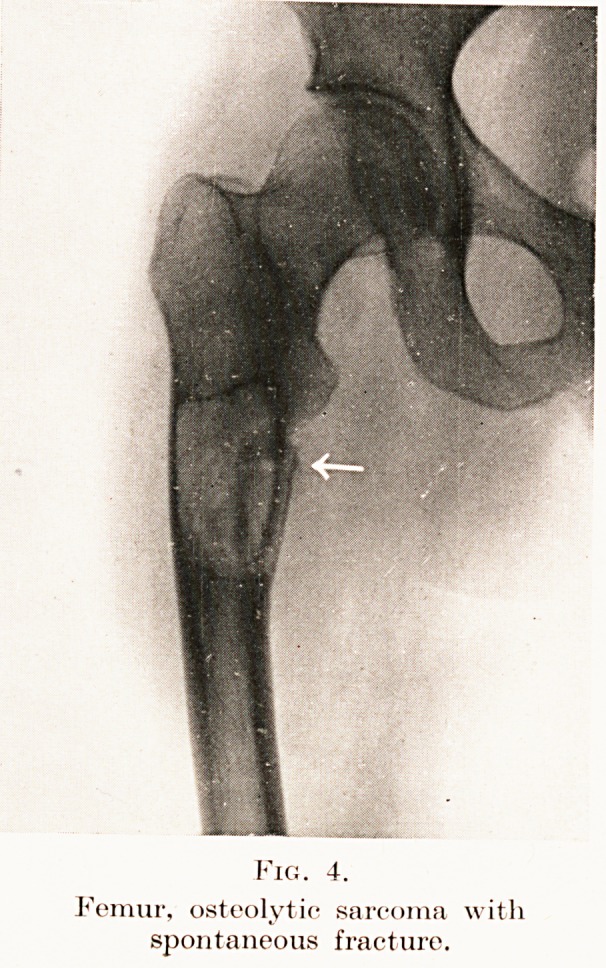


**Fig. 5. f6:**
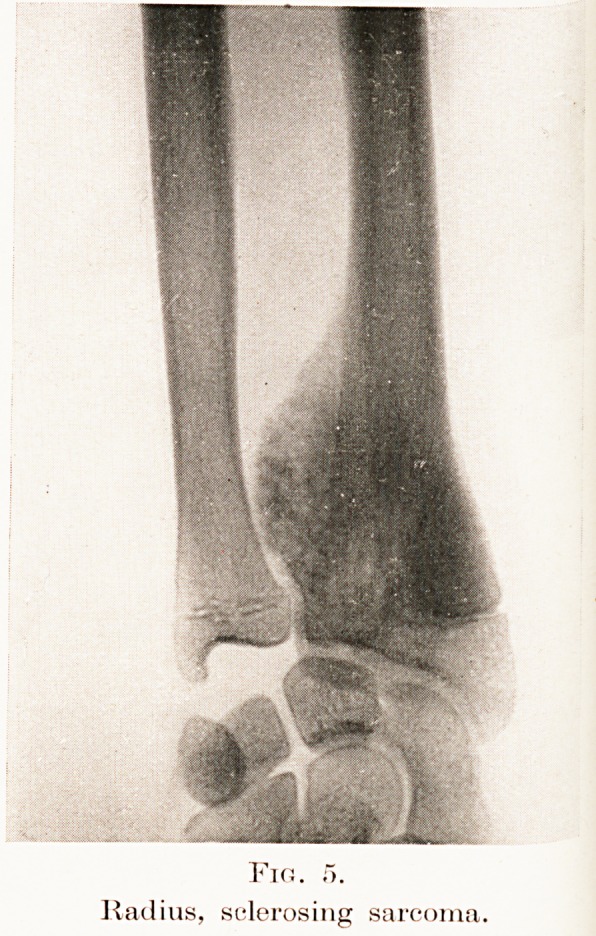


**Fig. 6. f7:**